# A Table of the Greatest, Least, and Mean Heights of the Thermometer and Barometer, and of the Fall of Rain in London in 1780

**Published:** 1782

**Authors:** 


III. *A TABLE of the Greatest, Least, and Mean Heights of the Thermometer and Barometer, and of the Fall of Rain in London in 1780. (From the* Philosophical Transactions, Vol. 71, Part I.)

| 1780. | Thermometer without. | | | Thermometer within. | | | Barometer. | | | Rain. |
|---|---|---|---|---|---|---|---|---|---|---|
| | Greatest Height. | Least Height | Mean Height. | Greatest Height. | Least Height. | Mean Height. | Greatest Height. | Least Height. | Mean Height. | Inches. |
| January | 47,0 | 20,0 | 31,9 | 40,5 | 24,5 | 33,6 | 30,35 | 28,59 | 29,77 | 0,692 |
| February | 53,5 | 20,0 | 37,8 | 49,0 | 31,0 | 38.4 | 30,52 | 29,08 | 29,91 | 0,858 |
| March | 59,0 | 34,5 | 51,4 | 56,5 | 36,0 | 49,9 | 30,45 | 29.38 | 29,91 | 1,189 |
| April | 65,5 | 33,0 | 46,7 | 65,0 | 38,0 | 48,0 | 30,17 | 28,81 | 29,65 | 2,739 |
| May | 84,5 | 45,0 | 59,7 | 74,5 | 51,0 | 60,4 | 30,28 | 29,38 | 29,94 | 0,822 |
| June | 84,5 | 48,0 | 62,9 | 76,0 | 50,0 | 63,2 | 30,37 | 29,79 | 30,01 | 0,852 |
| July | 82,0 | 54,0 | 66,4 | 78,5 | 61,0 | 69,4 | 30,34 | 29,59 | 30,05 | 1,602 |
| August | 83,5 | 58,5 | 69,2 | 76,5 | 62,0 | 68,3 | 30,20 | 29,85 | 30,05 | 0,485 |
| September | 84,0 | 48,0 | 61,3 | 79,0 | 56,0 | 65,2 | 30,20 | 29,14 | 29,83 | 2,903 |
| October | 66,0 | 41,5 | 52,3 | 65,0 | 46,0 | 53,9 | 30,29 | 28,66 | 29,69 | 2,356 |
| November | 52,0 | 26,0 | 42,0 | 53,0 | 36,0 | 43,6 | 30,47 | 28,94 | 29,85 | 2,505 |
| December | 50,0 | 24,0 | 38,5 | 46,5 | 32,0 | 39.8 | 30,55 | 29,69 | 30,29 | 0,319 |
| Whole Year | | | 51,7 | | | 52,8 | | | 29,91 | 17,313 |

